# 3 in 1: Manifestations of Multiple Endocrine Neoplasia Type 2B on Imaging

**DOI:** 10.1210/jcemcr/luaf004

**Published:** 2025-01-24

**Authors:** Victoria Belcher, Tyler Hinshaw, James Field, Adnan Haider

**Affiliations:** Department of Internal Medicine, West Virginia University School of Medicine, Morgantown, WV 26505, USA; Department of Internal Medicine, West Virginia University School of Medicine, Morgantown, WV 26505, USA; Section of Endocrinology and Metabolism, Department of Internal Medicine, West Virginia University School of Medicine, Eastern Division, Martinsburg, WV 25401,USA; Section of Endocrinology and Metabolism, Department of Internal Medicine, West Virginia University School of Medicine, Morgantown, WV 26505, USA

**Keywords:** multiple endocrine neoplasia, adrenal mass, ganglioneuromatosis, chronic megacolon

## Image Legend

**Figure 1.**
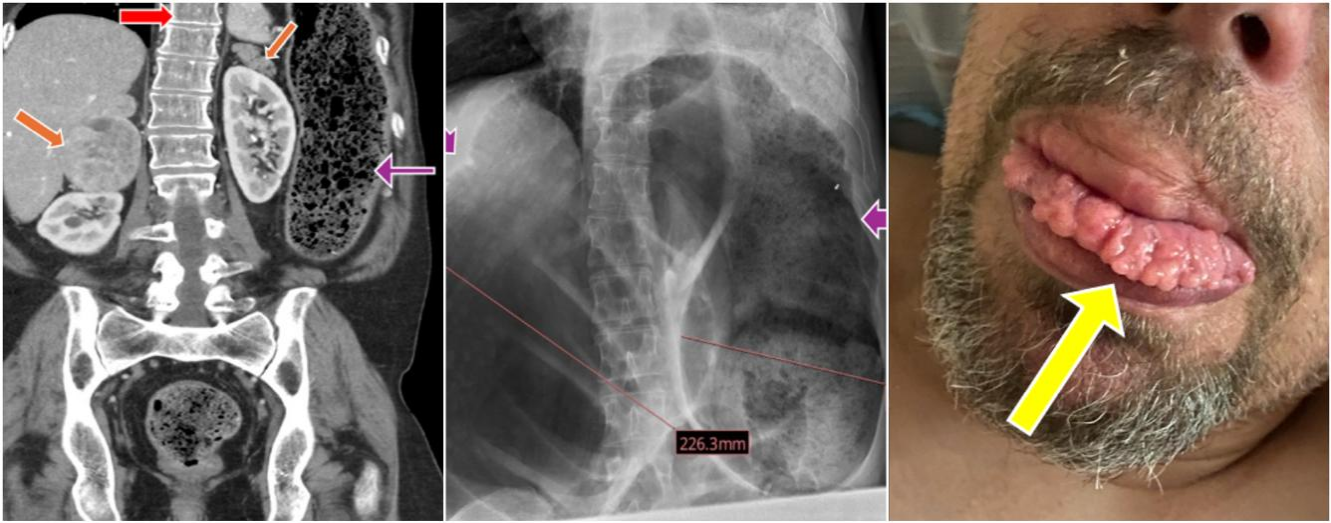
CT scan of the abdomen shows bilateral adrenal masses, lateral scoliosis and dilated colon on abdomen X-ray & mucosal neuromas on the tongue.

A 50-year-old male with a history of medullary thyroid cancer, treated with total thyroidectomy at age 22, and a known pathogenic variant of the *RET* protooncogene, presented with abdominal distention and inability to pass flatus. Imaging, including abdominal computed tomography scan and supine X-ray, showed bilateral adrenal masses (Fig. 1 brown arrows), massive colonic dilation (Fig. 1 purple arrows), and lateral scoliosis (Fig. 1 red arrow). Physical examination noted marfanoid habitus, mucosal neuromas (Fig. 1 yellow arrow) of the lips, and a tympanic abdomen. Two years prior, plasma-free normetanephrine and plasma-fractionated metanephrines were significantly elevated. Despite recommendations for bilateral adrenalectomy, the patient declined surgery until this presentation. Following alpha-adrenergic blockade and hydration, he underwent bilateral adrenalectomy, total colectomy, splenectomy, and ileostomy. Pathology revealed diffuse colonic ganglioneuromatosis, 6 cm right adrenal pheochromocytoma, and a 1.4 cm left adrenal pheochromocytoma. Postoperatively, plasma metanephrines normalized.

Multiple endocrine neoplasia type 2B syndrome can present as chronic constipation with intermittent diarrhea, secondary to chronic megacolon. Ganglioneuromatosis causes enteric neural dysfunction, leading to multiple gastrointestinal problems including chronic constipation, achalasia, volvulus, or diverticulum formation [1]. Elevated catecholamines exacerbate reduced colonic tone, leading to toxic megacolon. Multiple endocrine neoplasia type 2B patients commonly present with gastrointestinal problems, with some pediatric cases presenting with intestinal volvulus and chronic megacolon in all ages [2].
